# Response to “Normal spirometry equates to normal impulse oscillometry in healthy subjects”

**DOI:** 10.1186/s12931-021-01721-z

**Published:** 2021-04-20

**Authors:** Liang-Yuan Li, Tian-Sheng Yan, Jing Yang, Yu-Qi Li, Lin-Xi Fu, Lan Lan, Bin-Miao Liang, Mao-Yun Wang, Feng-Ming Luo

**Affiliations:** 1 Department of Respiratory and Critical Care Medicine, West China School of Medicine and West China Hospital, Sichuan University, No. 37 Guoxue Alley, Chengdu, 610041 China; 2 Department of Critical Care Medicine, West China School of Medicine and West China Hospital, Sichuan University, Chengdu, 610041 China

We thank B. Lipworth and R. Chan for their constructive comments on our article “Impulse oscillometry for detection of small airway dysfunction in subjects with chronic respiratory symptoms and preserved pulmonary function” [1]. We really appreciate that they mentioned the pragmatic abnormal IOS values in their clinic. The cutoff values obtained in our study are lower than the abnormal values in their study and some others’ [2–4]. But the target population in the above studies were patients who were diagnosed with COPD or asthma. In contrast, our subjects were those who do not meet the pulmonary function criteria of COPD or asthma, only presenting small airway dysfunction (SAD) in spirometry. A large multistage stratified sampling survey in China showed that more than 20% of Chinese suffered from such pure SAD with preserved pulmonary function [5]. Our object was to explore a better screening tool targeting such a population. Besides, the differences in race and age would influence the results when the absolute IOS values rather than the predicted percent values were used. The biggest limitation of our study was the small sample size, especially for the size of SAD population, which is only 42. Future studies with large sample size and the application of IOS predicted percent values are needed.

We agree with the authors regarding the false positive possibility of spirometry. Up to now, there is still not a gold standard for SAD. On account of the effort dependence, spirometry is not objective enough to evaluate SAD. Prospective studies should use more objective measurements as the standard.

All in all, as exploratory research, our major objective was to confirm the better sensitivity and correlation with symptoms of IOS compared to spirometry, rather than to find a definite cutoff value. The early detection of SAD facilitates early getting out of risk factors such as cigarettes. And we usually do not give too many clinical or drug interventions to these people. Which is the most important is that the results of spirometry or IOS should not be considered alone but combined with symptoms and images.

## Data Availability

Not applicable.

## Abbreviations

IOS: Impulse oscillometry
COPD: Chronic obstructive pulmonary disease
SAD: Small airway dysfunction
